# The role of triple therapy and therapy sequence in treatment of BRAF-mutant metastatic melanoma. Response to overall survival with first-line atezolizumab in combination with vemurafenib and cobimetinib in BRAFV600 mutation-positive advanced melanoma (IMspire150): second interim analysis of a multicentre, randomised, phase 3 study

**DOI:** 10.1186/s12967-023-04391-1

**Published:** 2023-08-05

**Authors:** Reinhard Dummer, Michèle Welti, Egle Ramelyte

**Affiliations:** 1 Department of Dermatology, University Hospital Zurich, Zurich, Switzerland; 2 Faculty of Medicine, University of Zurich, Zurich, Switzerland

**Keywords:** BRAF mutant melanoma, Triple, Immunotherapy, Targeted therapy

## Abstract

Novel therapies have achieved unprecedented benefit in survival of advanced melanoma patients. While immunotherapy (ICI) can be administered independent of mutational status, BRAF and MEK kinase inhibitors represent another effective treatment option for patients with BRAF mutant melanoma. Given the benefits these therapies demonstrate, the natural instinct was to combine. Three studies have investigated the benefit of combination of ICI using anti-PD-1 or anti-PD-L1 antibody and targeted therapy (TT) with BRAF and MEK inhibitors over TT and placebo. Among these studies, statistically significantly superior duration of response was observed, however overall and progression-free survival were only numerically superior, if at all. One triple combination was approved for BRAF mutant metastatic melanoma; however, the expected synergistic effect of triple therapy could not be universally confirmed and the observed benefits with triple seem to depend on statistical considerations rather than a biological reason. As patients with BRAF mutant melanoma have both ICI and TT as their first-line treatment options, the question whether the sequence matters was addressed. Two prospective trials compared first-line ICI, followed by TT at progression, or vice-versa, with additional “sandwich” approach (8 weeks of TT followed by ICI until progression, then TT again) in the Secombit study. The benefit of first-line ICI was demonstrated in both studies with Secombit study showing the “sandwich” approach to have similar effect. Current data advices for immunotherapy based regiments in patients with BRAF mutant melanoma or, possibly, sandwich approach. Whether triple therapy is superior to ICI monotherapy still needs to be addressed considering not only efficacy, but also safety.

## Introduction

Immunotherapy and targeted therapy have demonstrated unprecedented efficacy and have significantly improved the outcome of patients with metastatic melanoma. The major advantage of immunotherapy (IT) is the long-term benefit even if the treatment is discontinued. The strength of targeted therapy (TT) is the immediate response of most patients treated that can ameliorate critical disease related symptoms in the individual patient. Based on these major differences between the two therapy types, there was the clear promise that the combination of both might keep the advantages of every single combination partner [1].

The hypothesis of potential synergies of TT/IT combinations has been based on preclinical data, demonstrating that the activation of the mitogen-activated protein kinase (MAPK) pathway causes an immunosuppressive microenvironment including the presence of cytokines dimishing T-cell activity, decreased expression of melanoma differentiation antigens (MDA) and surface human leukocyte antigens class I (HLA-I). This results in reduced activity and functionality of tumor specific cytotoxic T cells [2]. It is speculated that BRAF inhibitors mediate immune sensitizing effects including enhanced antigen presentation.

## Synergistic effect of triple with immunotherapy and targeted therapy?

Consequently, three major prospective randomized clinical trials have been initiated and conducted which are in the follow up phase now.

IMspire150 was the first phase III study to investigate combination of IT and TT (triple therapy) in stage IIIC-IV BRAFV600-mutant melanoma [3]. It was initiated given the promising results from the phase 1b study, in which the induction with vemurafenib and cobimetinib was followed by the addition of atezolizumab after the first treatment cycle was tolerable, detected promising immunological alterations in biopsies and showed initial efficacy [4]. In the IMspire150 study, 514 treatment-naive patients patients were randomized in a 1:1 ratio to receive a triple combination of PD-L1 inhibitor atezolizumab with the BRAF inhibitor vemurafenib and the MEK inhibitor cobimetinib versus vemurafenib and cobimetinib. Of note, the dose of vemurafenib was reduced from 960 mg bid to 720 mg bid upon addition of atezolizumab in the second treatment cycle. The primary endpoint of IMspire150 was investigator-assessed progression-free survival (PFS). In the 1st analysis, overall response rate (ORR) was similar between the two groups (66.3% vs. 65%) with similar detection of complete response (CR, 15.7% vs. 17.1%, respectively). However, patients treated with triple therapy demonstrated a longer median duration of response (mDoR) than vemurafenib and cobimetinib alone (21 vs. 12.6 months, respectively). The PFS curves showed separation after 7 months of treatment, suggesting that addition of atezolizumab prolongs treatment benefit in BRAF-mutant metastatic melanoma.

Ascierto et al. have just now published data from a longer follow-up including overall survival data for IMspire150 [5]. The results confirm a significant improvement of PFS by the addition of this anti-PDL1 antibody. The improvement still is not dramatic (hazard ratio 0.79), as well as the results for overall survival. The difference between the two arms is not significant with a hazard ratio of 0.84.

We need to stress that these advantages in important outcome parameters are accompanied by additional adverse events and discomfort for the patients due to the repeated infusion procedures. In this study, the subgroup analysis is also interesting, as we see that the benefit from the triplet is preferentially found in the patient population with good prognostic features including normal lactate dehydrogenase (LDH) and limited number of metastases. Patients of advanced age seem to profit more than younger patients. The research community would have preferred to see the benefit of the triplet therapy mainly in high-risk patients characterized by elevated LDH and multiple organ involvement, because these patients have a very high medical need. The triple combination of atezolizumab, vemurafenib and cobimetinib was also used in patients with brain metastases [6] with efficacy results that appear very similar to the efficacy of TT alone [7]. Similar results have been reported for other trials.

Keynote-022 is a phase II study that investigated another triple combination with anti-PD-1 antibody pembrolizumab combined with BRAF inhibitor dabrafenib and MEK inhibitor trametinib. Hundred-twenty patients that were randomized at 1:1 ratio to receive pembrolizumab, dabrafenib and trametinib, or dabrafenib and trametinib. The primary endpoint of Keynote-022 was PFS; secondary endpoints were ORR, DoR and overall survival (OS). A numerical improvement in PFS and OS was reported in the triplet arm, compared to control arm, however, not statistically significant and the trial did not meet the primary endpoint [8]. The last update of Keynote-022 with a median follow up of 61.2 months (range 50.7–67.5), reported a longer median PFS in the triple arm reaching 17 months (95% confidence interval (CI) 11.3 to not reached (NR)), compared to 9.9 months (95% CI 6.7 to 15.6) in the dabrafenib and trametinib arm. The hazard ratio (HR) for progression was 0.46 (95% CI 0.29 to 0.74) [9]. The median OS with 46.3 months (95% CI 23.9-NR) was also longer in the triple arm compared to 26.3 months (95% CI 18.2–38.6) in the doublet arm. The exploratory subgroup analysis for PFS suggested that patients that are < 65 years, of male gender, have an eastern cooperative oncology group (ECOG) performance status of 0 and those with elevated LDH level at baseline were more likely to profit from the triple therapy. However, none of these factors demonstrated significant impact on prolonged OS. In the Keynote-022, the patients who received the doublet therapy showed higher ORR than patients in the triple therapy arm (65% with triple vs. 72% with double), however more complete responses were detected in the triple arm (20% with triple vs. 15% with double). The mDoR was significantly longer in the triplet arm compared to the doublet arm (30.2 months vs. 12.1 months; HR 0.32; 95% CI 0.17–0.59). Dabrafenib and trametinib showed better safety profile with triple therapy leading to higher incidence of high-grade immune-related adverse events. Despite this, drug exposure to BRAF/MEK inhibitors was higher in the triple therapy arm, compared to the double group (12.4 vs. 9.1 months).

Another study that investigated the combination of IT and TT in patients with unresectable stage IIIC-IV cutaneous *BRAF*V600-mutant melanoma was is COMBI-i. In this phase 3 study, patients were randomized to receive a triple combination with anti-PD-1 antibody spartalizumab, dabrafenib and trametinib, or the combination of dabrafenib with trametinib and placebo (double therapy) [10]. The primary endpoint of COMBI-I study was investigator-assessed PFS, OS was among the secondary endpoints. Along with IMspire150, the COMBI-I study did not meet the primary endpoint of significantly prolonged PFS. However, the results of this study were in concordance to findings the Keynote-022 trial [11]: after a mFU of 27.2 months (IQR 25.4–29.0 months), triple therapy demonstrated numerically superior mPFS (16.2 months (95% CI 12.7–23.9 months)) compared double therapy (12.0 months (95% CI 10.2–15.4 months)) and a HR for progression of 0.82 (95% CI 0.66–1.03; *p* = 0.042; one-sided). The subgroup analysis for the PFS suggested that patients with ≥ 3 metastatic sites (*p* = 0.03) and a sum of lesion diameters ≥ 66 mm at baseline (*p* = 0.007) benefit more from the triple than double therapy. The ORR was similar between the two treatment groups (69% vs. 64% with triple and double, respectively), with similar proportion of patients with CR (20% vs. 18%). Moreover, at the time of report, the mDoR was not reached in the triple arm (95% CI 18.6 months—NR), compared to 20.7 months (95% CI 13.0—NR) in the double arm. In the landmark analysis of 3-year OS with a mFU of 42.8 months, the median OS (mOS) was still not reached in the triple arm, but demonstrated mOS of 40.4 months in the double arm with HR for death of 0.79 (95% CI 0.62–1.03). Similarly to the other studies, the subgroup analysis suggested that patients with ECOG PS 1, age ≥ 65 years, negative PD-L1 status, sum of lesion diameters ≥ 66 mm at baseline and metastatic sites ≥ 3 benefit more from the triple therapy with a prolonged OS, compared to double therapy [12].

In summary, we can conclude that the triple approach did not result in a convincing benefit in the first line setting in mostly previously untreated advanced melanoma patients. Therefore, it was essential to understand how the two major treatment strategies should be sequenced to optimize the outcome in the BRAF mutant patient population.

## The implication of first-line therapy in BRAF mutant melanoma

Two randomized studies, SECOMBIT and DREAMseq, investigated the optimal first-line treatment in patients with BRAF-mutant unresectable melanoma.

SECOMBIT is a phase 2 study, in which patients were randomized to receive first-line encorafenib/binimetinib (BRAF inhibitor, MEK inhibitor) followed by ipilimumab/nivolumab upon disease progression (arm A), first line ipilimumab/nivolumab followed by encorafenib/binimetinib upon disease progression (arm B) and to “sandwich” arm, where 8-week induction phase with encorafenib/binimetinib was followed by a treatment switch to ipilimumab/nivolumab until disease progression and then back to encorafenib/binimetinib upon further progress (arm C) [13]. The primary endpoint of SECOMBIT study was 2-year OS, secondary endpoints were PFS on the first line therapy and total PFS, defined as the time to the second progression. Despite the small study population, the mOS was not reached in any of the study arms after an mFU of 32.2 months. The study was not powered to compare the three treatment arms, however, the landmark 2-year OS rate was higher in arm B (73%) compared to arm C (69%) and arm A (65%), and, respectively, 62%, 60% and 54% at 3-year landmark OS [13]. While treatment sequence in Arm A and B is corresponds to common clinical practice, arm C was based on translational data, which suggested transient nature of BRAF/MEK induced immunomodulatory effects. Clinical and preclinical data support the hypothesis that acquired resistance to TT, mediated by a tumor microenvironment lacking functional dendritic cells and showing immunosuppressive properties, can induce a cross-resistance to ITs [14]. Indeed, short exposure to TT (arm C) demonstrated a clinical benefit compared to TT exposure until progression (arm A), but did not show any superior treatment responses compared upfront IT (arm B). These data are in line with previous retrospective reports in real-life patients [15].

Another study to have investigated the treatment sequence in patients with BRAF-mutant unresectable melanoma was DREAMseq. In this phase III study, treatment-naive patients were randomized to receive either upfront IT with ipilimumab/nivolumab (arm A) or upfront TT with dabrafenib/trametinib (arm B) with crossover to the alternate therapy at disease progression [16]. As in the SECOMBIT study, the primary endpoint was landmark 2-year OS rate. Upfront IT with ipilimumab/nivolumab (arm A) demonstrated superior 2-year OS rate with 71.8%, compared to 51.5% in patients starting with TT in arm B (*p* = 0.01). The observed survival data was in line with the 2-year OS rate from the SECOMBIT study, although upfront TT (arm B) showed somewhat lower performance in DREAMseq. In line with the previous data, treatment with ipilimumab/nivolumab after progression on first line BRAF/MEK inhibitors resulted in lower response rates than upfront ipilimumab/nivolumab.

## Conclusion

These recent clinical trials have explored several aspects of sequencing and combining the principle therapeutic approaches for BRAF mutated melanoma. The sponsors and the investigators must be congratulated for this network of sophisticated clinical trials that have included more than 1200 patients. The result is decent evidence that starting with immunotherapy first is the appropriate strategy. In patients that need immediate tumor reduction, a short-term use of targeted therapy might be considered with a switch to combination immunotherapy after 8 to 12 weeks. Some of us have expected another outcome, but this is the justification of clinical research: generate evidence instead of gut feeling!

## Data Availability

For data and material please refer to original publications with corresponding references. No unique data or material was produced or reported on in this commentary.

## Abbreviations

IT: Immunotherapy
TT: Targeted therapy
MDA: Melanoma differentiation antigens
DoR: Duration of response
mDoR: Median duration of response
PFS: Progression free survival
mPFS: Median progression free survival
OS: Overall survival
mOS: Median overall survival
FU: Follow up
mFU: Median follow up
NR: Not reached
ORR: Overall response rate
HR: Hazard ratio
MAPK: Mitogen-activated protein kinase
CR: Complete response
ECOG: Eastern cooperative oncology group
LDH: Lactate dehydrogenase
